# Nonsurgical Facial Contouring With a Novel Radiofrequency and Vacuum Device: A Multicenter Retrospective Study With 33 Patients

**DOI:** 10.1111/jocd.70127

**Published:** 2025-04-02

**Authors:** Mimi Ehrenraich, Ety Wolff, Suzy Roffe Grumer, Olalla Calvo Lozano, Reyna Vargas, Inna Belenky

**Affiliations:** ^1^ Mimi Ehrenraich Aesthetic Clinics Haifa Israel; ^2^ Wolff Aesthetics Center Herzliya Israel; ^3^ Arival Ltd. Tel‐Aviv Israel; ^4^ Sinclair London UK

**Keywords:** facial aging, facial contouring, radiofrequency, skin laxity, vacuum

## Abstract

**Background:**

The aging process reflects decreased tissue elasticity and a rearrangement of subcutaneous volume, resulting in superficial wrinkles and three‐dimensional changes in skin topography. Radiofrequency (RF)‐based approaches have become increasingly adopted in recent decades due to their relatively short downtime and significantly reduced risk of adverse effects.

**Aims:**

The aim of this study was to evaluate the safety and efficacy of the V‐FC handpiece through a multicenter retrospective study as well as ex vivo and in vivo analyses.

**Methods:**

The V‐FC handpiece was used once a week for a treatment course of 3–6 sessions. The clinical evaluation of 33 patients included demographics, BMI, assessment of skin laxity, and rate of improvement. An ex vivo study with porcine tissue assessed the penetration depth of RF, while device performance was evaluated in an in vivo animal histological analysis.

**Results:**

No significant adverse effects were recorded during the treatment course or at short‐ and long‐term follow‐ups (1, 3, and 52 months). The average skin laxity grade baseline was 34% lower at the end of the treatment course, with the average rate of improvement assessed as moderate. The ex vivo minimal and maximal penetration depths were 3.2–11.3 mm (±10%). The in vivo histopathological analysis showed no signs of acute heat injury at any treatment setting, including the maximum RF power. Additionally, staining showed a power‐related progressive proliferation of fibroblasts, elastin fibers, and neocollagenesis, which was significantly higher 4 weeks after treatment.

**Conclusions:**

The new RF device demonstrates an effective treatment for facial contouring with a proven short‐ and long‐term high safety profile.

## 1 Introduction

The aging process of the human face reflects the cumulative effects of gravity, decreased tissue elasticity, and rearrangement of subcutaneous volume, resulting in both superficial textural wrinkling of the skin and changes in the three‐dimensional topography of the underlying structures [1, 2].

Radiofrequency (RF)‐based devices use the tissue's natural resistance to electrical currents to transform RF energy into thermal energy in the tissue [3]. These devices are generally used for various skin conditions, including skin tightening and body contouring, due to RF thermal capability to produce collagen remodeling, fibroblast stimulation, increased blood circulation, and reduced fat volume [4, 5, 6, 7].

The new V‐FC handpiece incorporates bipolar channeling optimized radiofrequency energy (CORE) technology and vacuum suction. CORE allows targeting different tissue depths due to its four different frequencies. The integrated vacuum suction is intended to improve blood microcirculation [8], lymphatic drainage [9], fibroblast stimulation, and proliferation [10, 11], generating a synergetic effect on the tissue. These features position the new device as a higher‐ranking technology compared with RF devices reported in the scientific literature for skin tightening and contouring. Most recent RF devices work with a single RF or dual without vacuum [12, 13] or incorporate rotation technology to provide active massage [14, 15].

The aim of this study was to evaluate the safety and efficacy of a new RF device combining RF and vacuum technology. The device performance was evaluated by a multicenter retrospective study as well as by an ex vivo thermal camera and in vivo histological evaluation.

## 2 Methods and Materials

### 2.1 Data Collection

Thirty‐three patients who were treated in three separate clinical centers (Mimi Ehrenraich Aesthetic Clinics, Israel; Wolf Aesthetics Center, Israel; and Sinclair in‐house), between November 2019 and January 2024, were retrospectively evaluated. Only patients with full clinical data records, “Before” and “After” images, and facial aging with skin laxity and/or facial local fat accumulation were included.

### 2.2 Handpiece Description

V‐FC is a bipolar RF handpiece (Viora Ltd., by Sincalir, UK) that utilizes patented CORE technology [4, 16] (Patent No. US 10864305 B2) and vacuum suction. Channeling optimized radiofrequency energy uses three different RF frequencies (0.8, 1.7, and 2.45 MHz) which can be released individually or combined for customized treatments targeting different tissue depths. The handpiece incorporates an integrated IR thermometer for continuous skin temperature monitoring and skin impedance measurement. The operator may adjust the handpiece's RF power (up to 35 W), vacuum pressure intensity, and RF frequencies.

### 2.3 Treatment Regimen

The treatment course involved a total of 3–6 sessions performed once a week (7 ± 1 day) using the V‐FC handpiece on one of the V‐SERIES platforms (V10, V20, and V30; Viora Ltd., by Sincalir, UK), with each session lasting 15–20 min.

All treatment sessions were conducted after having obtained the patient's informed consent confirming their awareness of the treatment method, possible side effects, treatment expectations, and a health history questionnaire. The inclusion criteria included adult patients with mild to severe skin aging laxity and/or facial local fat accumulation. Patients with a history of one or more of the device's contraindications were excluded from the treatment. Some RF‐based contraindications were electric or metallic implants, skin diseases in the treatment area, pregnancy, an impaired immune system, and endocrine disorders. This study was conducted in compliance with the ethical guidelines of the 1975 Declaration of Helsinki.

All subjects maintained a stable weight (with weight fluctuations limited to −1.5 and +1.5 kg) during the entire treatment course and did not undergo any other aesthetic treatment that may directly or indirectly affect wrinkle depth, laxity, and skin texture or quality.

### 2.4 Clinical Assessment

The clinical assessments, including the patient's Body Mass Index (BMI) and photographs, were performed during the baseline session and 4 weeks after the last session.

- Safety—the clinical data records included any short‐term known and unknown adverse events, serious adverse events, or unexpected side effects that occurred during the treatment course and during the 4‐week follow‐up. All subjects were evaluated as well for long‐term adverse events via a follow‐up phone call or email during the retrospective data collection at the end of March 2024 (3–52 months follow‐up period).
- BMI—Body Mass Index was calculated by dividing each patient's recorded weight in kilograms during the baseline meeting by the square of their height in meters. Body height and weight were measured according to a standardized protocol, with participants standing without shoes and heavy outer garments [17]. The BMI change was calculated as follows: BMI at baseline and BMI at 4 weeks post the final treatment.
- Skin type—the skin type of each patient was classified with a rating of I–VI on the Fitzpatrick Scale [18].
- Clinical outcome assessment—the clinical outcome assessment was conducted via a blinded evaluator. The blinded evaluator received 30 sets of randomized “Before” and “After” images (one set per patient) without any indication of which photograph represented the “Before” image and which the “After” image. No additional data, such as age, sex, or skin type, or any identifying features (such as eyes) were shown in the sets of images.
  For each set of images, the blinded evaluator was asked to decide the following:
  1. Which photograph represented the “Before” and which was the “After” image?
  2. A skin laxity score for each image according to Table 1. Skin Laxity Grading Scale allows visual examination of the subject, noting the levels of severity. In this scale, the lower the score, the better the perceived outcome.
  3. The rate of improvement is based on a subjective 4‐point scale, according to Table 2.

**TABLE 1:** Skin laxity grading scale [19].

Score	Classification	Description
0	None	No loose skin, toned, and firm skin with a smooth surface texture
1	Mild	Mildly loose skin, somewhat toned with a smooth surface texture
2	Moderate	Moderately loose skin, no deep tone, a few wrinkles, and creepiness on the skin surface
3	Severe	Very loose skin without underlying tone, multiple wrinkles and creepiness on the skin surface, and skin distinct from underlying subcutaneous tissue via palpation
4	Extreme	Prominent redundancy of skin without underlying tone, severe wrinkling and creepiness on the skin surface

**TABLE 2:** Rate of improvement.

Score	Rate of improvement	Description
0	No improvement	No visible changes in skin laxity or face contour. Skin remains loose with no definition or firmness
1	Mild improvement	Slight enhancement in skin firmness and contouring. Minimal reduction in sagging, but changes are barely noticeable
2	Moderate improvement	Noticeable improvement in skin tightness and face contouring. Sagging is reduced, and the face appears more defined
3	Significant improvement	Dramatic enhancement in skin firmness and contour. Clear lifting effect with well‐defined contours and minimal sagging

### 2.5 Ex Vivo Depth Penetration Measurement

An ex vivo experiment using porcine tissue evaluated V‐FC handpiece penetration depth capabilities at different settings using a thermal camera. Porcine tissue was stored at room temperature of 15°C–20°C, and the superficial skin was removed by surgical scalpel. Following the application of a thin layer of glycerin gel on the porcine tissue, a 2‐min V‐FC treatment was performed at maximum RF power (35 W) in all RF frequency modes (0.8, 1.7, and 2.45 MHz and a combination of all three frequencies) and all vacuum levels (1–4). The tissue's temperature change was recorded and measured by a Ti200 infrared thermal imaging camera (Fluke Corp, Washington) that was perpendicularly positioned to obtain cross‐sectional temperature maps. The thermal videos and images were analyzed using Fluke Connect software (FlukeConnect Application, v.1.1.547, US). An isothermal area of 5°C under the maximum measured temperature was set to calculate the penetration depth of the heat inside the porcine tissue, while ImageJ software (National Institutes of Health, Maryland) was employed to convert pixels from the thermal image into millimeters.

### 2.6 In Vivo Histological Evaluation

V‐FC performance was evaluated via an in vivo animal model histological evaluation. Five Bama Miniature Pigs (*Sus scrofa domestica*), 4–6 months old and 20.0–25.0 kg weight, were used in the study. Each animal was subjected to the standard V‐FC treatment protocol with different treatment settings: RF power (levels 1, 2, and 4), RF frequency mode (0.8, 1.7, and 2.45 MHz). All animals were observed for mortality and general condition at least once daily. The study was approved by the Qingdao Science Innovation Quality Testing Co. Ltd. Institutional Animal Care and Use Committee.

Ten duplicate skin biopsies were harvested from each exposure condition at 5 time points: Day 0 (immediately after), Day 3 (72 h), Days 7, 14, and 28. Harvested samples were histologically processed and analyzed for acute injury, collagen, and morphological changes in fibroblasts. Each sample was excised from the center of the activation site with full skin thickness (down to the underlying fat and subcutaneous tissue) using an 8‐mm biopsy punch. Harvested samples were individually fixed in 10% neutral buffered formalin (approximately 4% formaldehyde solution).

All biopsies were bisected perpendicular to the skin surface, and both halves were embedded in a paraffin block to be sectioned perpendicular to the skin surface. The histological sections were stained with hematoxylin and eosin (H&E) and Masson's trichrome.

Macroscopic evaluation for erythema, edema, exudate, crust, and bleeding of all activation sites not yet biopsied was performed prior to each tissue harvest.

### 2.7 Statistical Analysis

All statistical analyses were performed using Microsoft Excel 2021 (Office 365). Descriptive analysis was performed on the cohort, with the number of valid cases for each test, minimum and maximum values measured, average and standard deviation (SD), and percentage calculations recorded.

Correlations between two variables were calculated by the Pearson correlation coefficient (via CORREL function, “*r*”). If the correlation coefficient lies between ±0.50 and ±1, the correlation is strong (high degree). If the value lies between ±0.30 and ±0.49, the correlation is moderate (moderate degree). When the value lies between ±0.29 and ±0.05, the correlation is low (low degree). If the “*p*” value lies below 0.05, the correlation is significant. The “*p*” values for the Pearson correlation coefficient were calculated via regression test, with *α* = 0.05.

In addition, for the blinded evaluation of the change in the degree of skin laxity, the significance was evaluated with a one‐tailed *t*‐test with *α* = 0.05.

## 3 Results

### 3.1 Patient Profiles

The retrospectively collected records of thirty‐three patients included all females with Fitzpatrick skin phototypes ranging from II to IV (27% skin type II, 64% skin type III, and 9% skin type IV). The patients' age ranged between 23 and 80 years (average 51 ± 15). The calculated BMI of the patients ranged between 21 and 35 (average 26 ± 3). Elevan patients (33%) were in the range of normal “healthy weight” (BMI 18.5–24.9 kg/m^2^), 18 patients (55%) were in the “overweight” category (BMI 25–29.9 kg/m^2^) and four patients (12%) were in the “obese class I” (moderately obese) category (BMI 30–35 kg/m^2^) (Table 3).

**TABLE 3:** Blinded evaluation of 30 (of 33) B&A photo sets with skin laxity grade and rate of improvement.

Study ID	Skin type	Age	Number of treatments	BMI	Skin laxity before	Skin laxity after	Change in skin laxity	Rate of improvement
VFCW‐001	II	56	4	30	2	1	1	3‐Significant
VFCW‐002	III	72	3	27	2	1	1	3‐Significant
VFCW‐003	IV	70	4	25	2	1	1	3‐Significant
VFCW‐004	III	65	3	26	2	1	1	3‐Significant
VFCW‐005	III	64	3	24	3	2	1	2‐Moderate
VFCW‐007	III	51	6	35	3	2	1	2‐Moderate
VFCW‐008	II	31	4	24	1	0	1	1‐Mild
VFCW‐009	III	33	4	26	2	1	1	3‐Significant
VFCW‐010	II	37	4	25	2	2	0	0‐No
VFCW‐012	III	40	3	26	3	2	1	3‐Significant
VFCW‐013	III	57	4	24	3	3	0	0‐No
VFCW‐014	IV	48	3	32	3	2	1	3‐Significant
VFCW‐015	III	41	6	27	1	1	0	1‐Mild
VFCW‐016	III	67	5	28	2	1	1	3‐Significant
VFCW‐017	III	80	6	32	2	2	0	1‐Mild
VFCW‐018	II	23	3	22	1	0	1	1‐Mild
VFCW‐019	III	56	4	24	2	2	0	1‐Mild
VFCW‐020	III	51	5	24	0	0	0	1‐Mild
VFCW‐021	III	42	3	26	2	2	0	1‐Mild
VFCW‐022	III	62	4	25	2	2	0	1‐Mild
VFCW‐023	III	45	4	26	1	0	1	1‐Mild
VFCW‐025	III	50	4	28	2	2	0	1‐Mild
VFCW‐026	III	23	3	21	0	0	0	1‐Mild
VFCW‐027	II	67	4	26	2	1	1	2‐Moderate
VFCW‐028	III	52	3	27	1	1	0	0‐No
VFCW‐029	II	72	4	25	3	1	2	3‐Significant
VFCW‐030	II	70	6	26	4	1	3	3‐Significant
VFCW‐031	II	44	4	28	2	1	1	2‐Moderate
VFCW‐032	III	57	4	25	2	2	0	1‐Mild
VFCW‐033	II	36	4	25	1	1	0	1‐Mild

### 3.2 Clinical Outcomes

#### 3.2.1 Safety

All thirty‐three patient records did not include any significant or unexpected adverse reactions during, immediately after the treatment course, or 4 weeks after the last treatment. The immediate skin response posttreatment included the expected mild erythema and occasional edema, considered a positive endpoint.

The follow‐up phone calls or emails were conducted at the end of March 2024, representing a follow‐up period range of 3 months (after the last treatment at the beginning of January 2024) to 52 months (after the last treatment in November 2019). No long‐term or short‐term side effects were reported via follow‐up checkups.

#### 3.2.2 Performance

No change in the patient's weight or BMI was detected at 4 weeks post the final treatment compared to the baseline.

The treatment course was three sessions for 30% of the patients, four sessions for 49% of the patients, five sessions for 9% of the patients, and six sessions for 12% of the patients.

The blinded evaluator correctly identified 91% of the 33 sets of “Before” and “After” images, which were further evaluated in terms of improvement using a subjective 4‐point scale (Table 2). The average rate of improvement was ranked at 1.7 ± 1.1, representing moderate improvement.

The average baseline “skin laxity grade” was 1.9 ± 0.9, representing moderate skin laxity which was reduced by 34% to an average score of 1.3 ± 0.8 at the end of the treatment course, representing mild skin laxity according to the Skin Laxity Scale (Table 1).

A low degree of correlation was found between the demographic data, skin type, age, and BMI of the patients and the change in skin laxity grade. Also, a low degree of correlation was found between the number of treatments and the change in skin laxity grade (Table 4).

**TABLE 4:** Coefficients of correlation and *p*‐value between skin type, age, BMI, number of treatments, initial skin laxity, and clinical outcomes (change in skin laxity and rate of improvement).

	Change in skin laxity	Rate of improvement
Skin type	*r*(28) = −0.28, *p* = 0.13	*r*(28) = 0.11, *p* = 0.57
Age	*r*(28) = 0.28, *p* = 0.13	*r*(28) = 0.37, *p* = 0.04
BMI	*r*(28) = 0.13, *p* = 0.50	*r*(28) = 0.30, *p* = 0.11
Number of treatments	*r*(28) = −0.12, *p* = 0.53	*r*(28) = −0.02, *p* = 0.90
Initial skin laxity grade	*r*(28) = 0.55, *p* < 0.05	*r*(28) = 0.48, *p* < 0.05

A moderate positive correlation was found between the age and BMI of the patients and the rate of improvement. A low correlation was found between patient skin type and the rate of improvement score. No correlation was found between the number of treatments and the rate of improvement (Table 4).

The correlation of the initial skin laxity grade of the patients, assessed by the blinded evaluation, was a significant strong positive correlation with the change in skin laxity grade at the end of the treatment course and a significant moderate positive correlation with the rate of improvement (Table 4).

A significant moderate positive correlation was found between patient BMI and the number of treatments (*r*(28) = 0.40, *p* < 0.05).

The significance of skin laxity grade lowering was calculated as *p* < 0.05 (one‐tailed *t*‐test), confirming that the difference in skin laxity level after RF treatments has a high statistical significance.

### 3.3 Ex Vivo Depth Penetration Measurement

**FIGURE 1:**
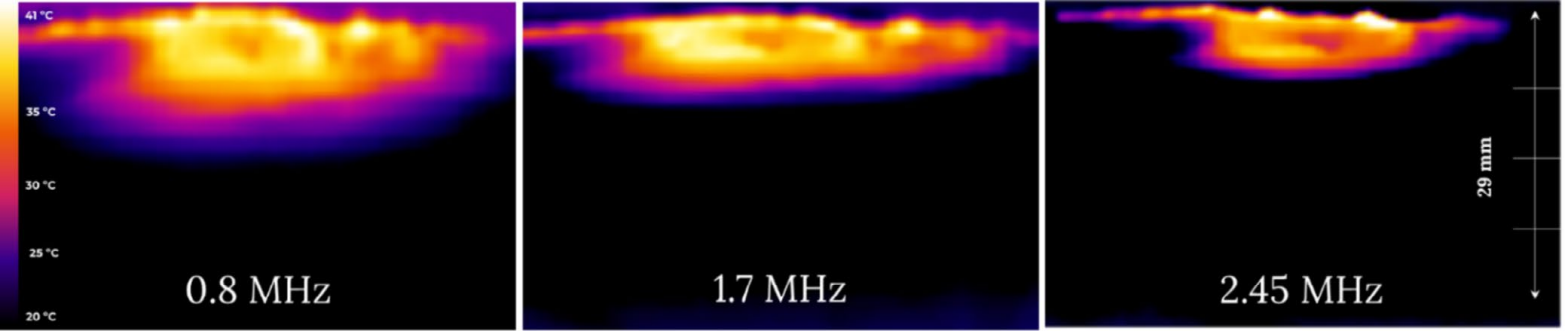
Ex vivo cross‐sectional thermal maps following RF treatment at 0.8, 1.7, and 2.45 MHz, with a constant RF power level of 35 W and vacuum suction.

The penetration depth of the V‐FC handpiece was measured ex vivo with a thermal camera. A total of 16 thermal maps output images, representing the different combinations of treatment settings (see example in Figure 1) were analyzed: four RF frequency modes (0.8, 1.7, and 2.45 MHz and a combination of all three frequencies) and four levels of vacuum suction at each RF frequency mode. The RF power was set to a constant maximum level of 35 W in all combinations.

The measured average minimum penetration depth was 3.2 mm (±10%) at the highest RF frequency (2.45 MHz) and the lowest vacuum level (level 1); and the average maximum penetration depth was 11.3 mm (±10%) at the lowest RF frequency (0.8 MHz) and the highest vacuum level (level 4) (Table 5). The measurements using the “multiple” mode with all 3 RF frequencies together were similar to those using 0.8 MHz: average penetration depth of 7.4–9.8 mm (±10%) vs. 7.1–9.2 mm (±10%), respectively.

**TABLE 5:** Ex vivo V‐FC penetration depth (in mm) measured via thermal camera (RF power 35 W).

RF frequency	Vacuum level^a^	Average depth (mm) ±10%
2.45 MHz	1	3.2–5.3
	4	5.1–8.1
1.7 MHz	1	6.2–8.2
	4	8.1–10.5
0.8 MHz	1	7.1–9.2
	4	9.7–11.3
Multiple (0.8, 1.7, and 2.45 MHz)	1	7.4–9.8
	4	9.7–11.5

^a^ The results measured with vacuum levels 2 and 3 are not shown.

### 3.4 In Vivo Histological Evaluation

No skin burns or epidermal shedding were observed immediately post V‐FC treatment. Furthermore, no visible injury or bleeding was detected on the skin at all observation points.

The histopathological evaluation showed no signs of acute heat injury at any treatment setting, including the maximum RF power of 35 W. Slight to mild acute inflammation (mainly monocytes) occurred in the subepidermal and perivascular regions and up to the superficial dermis. Tissue interstitial edema with neutrophil infiltration was predominant during the first week after treatment, followed by tissue repair (chronic inflammation mainly by lymphocytes) with fibrous tissue proliferation starting from the second week and fully completed after 4 weeks, with the highest histological response at the maximum RF power.

**FIGURE 2:**
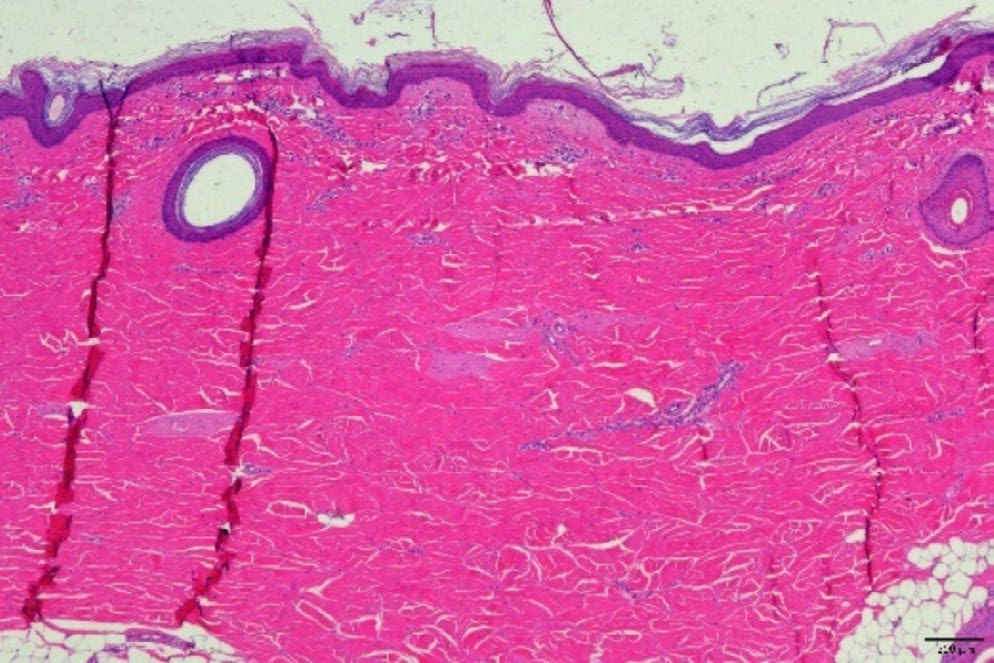
Histological section taken 4 weeks after V‐FC session at RF of 0.8 MHz and 35 W RF power, demonstrating normal histopathological findings in the skin.

H&E and Masson staining showed a power‐related progressive increase in fibroblasts (tissue proliferation) and neocollagenesis. The number of elastic fibers (filaments) in the dermis increased, and the number of fibroblasts significantly increased 4 weeks posttreatment (Figure 2).

## 4 Discussion

The clinical data obtained from both human clinical data and in vivo animal histopathological evaluation is evidence of V‐FC safety. No long‐term (3–52 months) or short‐term (1 month) side effects were reported via follow‐up checkups or documented in the treatment logs during the treatment course, supporting the prevailing consensus that RF‐based technology is a safe option for noninvasive facial contouring and skin tightening. The in vivo histopathological evaluation did not show any acute heat injury, even at the highest RF power. No textural skin damage such as burns, epidermal shedding, visible injury, or bleeding was observed immediately post‐RF treatment. The normal wound healing response began immediately after the RF session and was fully completed after 4 weeks.

Dermal remodeling by the V‐FC handpiece was clinically and histologically supported. Clinically, the indirect evaluation of circumferential reduction (as the outcome of connective tissue contraction) was measured by the skin laxity score. An average 34% reduction in the skin laxity score was assessed by the blinded evaluator, representing a reduction from “moderate” to “mild” in the skin laxity score. Histologically, connective tissue contraction was demonstrated in the animal in vivo experiment, where a power‐related progressive increase in fibroblast proliferation and neocollagenesis was detected posttreatment (Figure 2).

**FIGURE 3:**
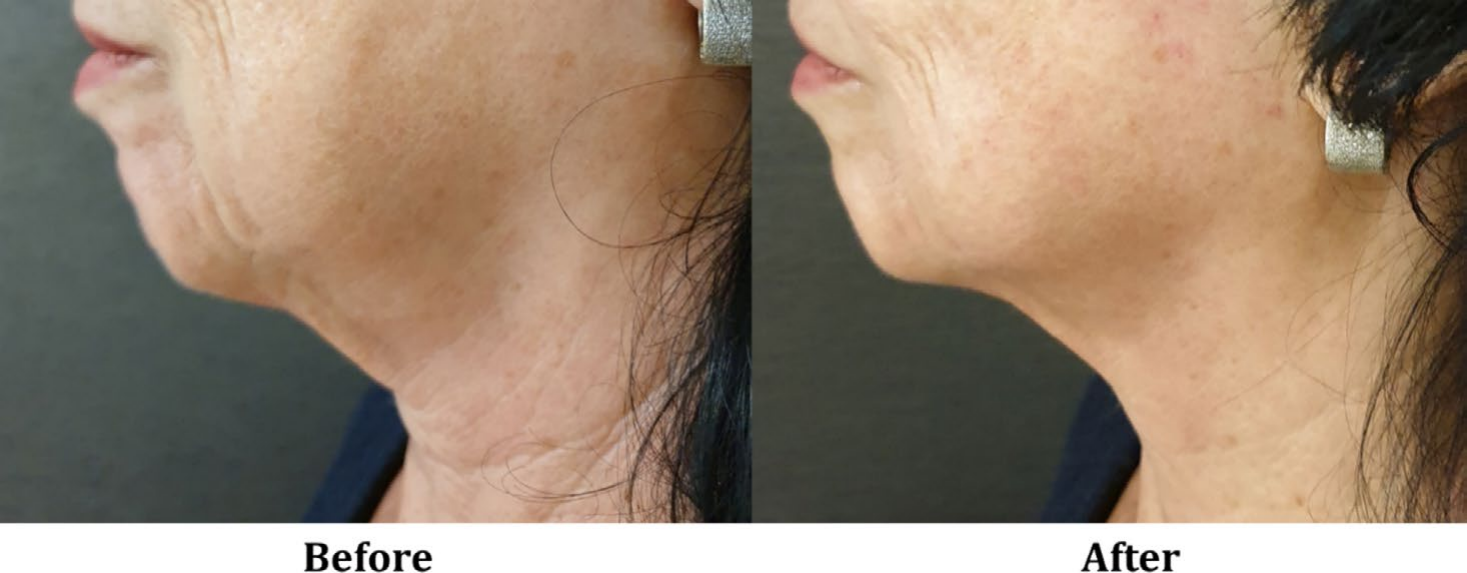
A 70‐year‐old female (Patient ID VFCW‐003), skin type IV, BMI 25: Before (left) and 4 weeks after (right) four treatment sessions with the V‐FC handpiece, with a “3—Significant” rate of improvement.

**FIGURE 4:**
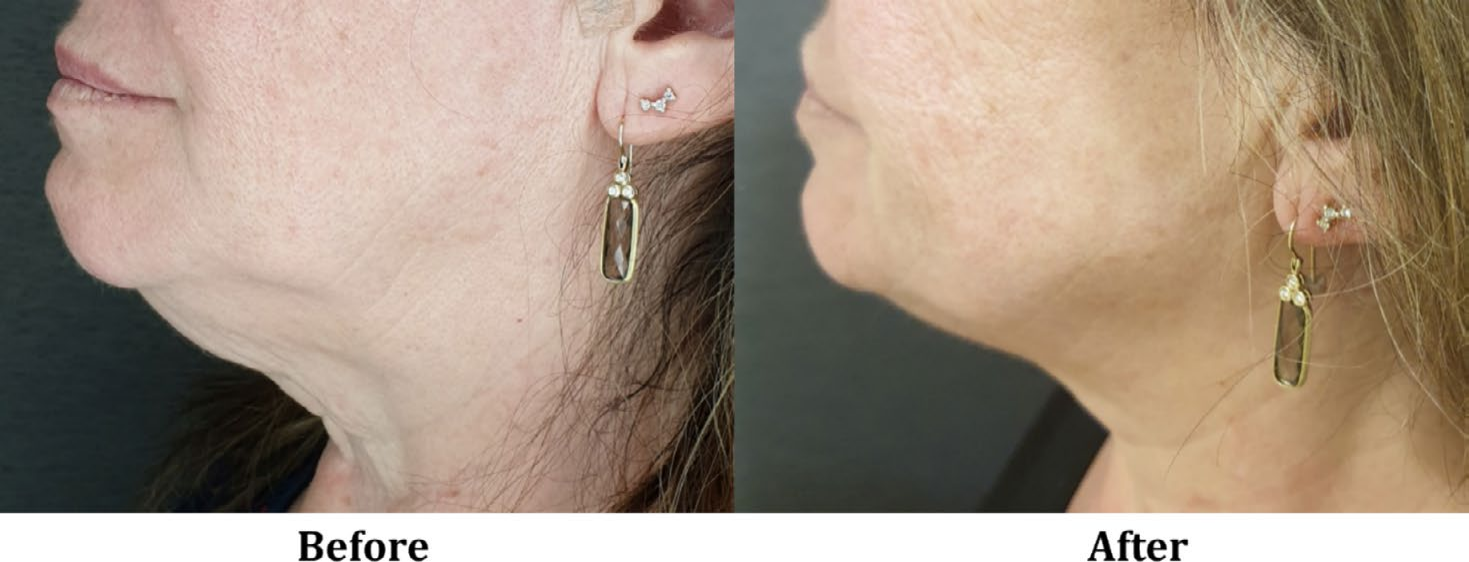
A 65‐year‐old female (Patient ID VFCW‐004), skin type III, BMI 26: Before (left) and 4 weeks after (right) three treatment sessions with the V‐FC handpiece, with a “3—Significant” rate of improvement.

The synergistic effect between the CORE RF‐based volumetric tissue heating and the vacuum‐based massage not only enhances dermal remodeling [8, 9, 10], but also facilitates better control over penetration depth. The ex vivo analysis using a thermal camera showed eight distinguishable levels of penetration depth, ranging from an average of 3.2 mm (±10%) up to 11.5 mm (Table 5). Similar behavior was detected in other RF‐based devices applicable with CORE technology and vacuum, but with a distinctive range of penetration depth since it is also influenced by the device size (mainly the distance between electrodes) [4, 16]. Additionally, the most recently reported RF devices have a single or dual RF frequency, with or without a massage system by rotation, limiting the versatility and personalization of the treatments. Therefore, leveraging the CORE technology, the practitioner is able to provide flexible, precise, and tailor‐made treatments for both facial contouring and circumferential reduction, as seen in Figures 3 and 4.

Since no change in the weight and BMI of the patients was detected at the end of the treatment course, and the patients did not receive any additional aesthetic treatment that may affect skin quality, laxity, or fat accumulation, the clinical outcomes assessed by the blinded evaluator can be attributed solely to the RF device.

The relatively short treatment course (3–6 sessions) demonstrated a satisfactory response, resulting in a moderate improvement outcome. In RF treatments, the variation in the number of sessions between individuals is influenced by several factors, including skin condition and severity, size and location of the treatment area, individual response to RF energy, and treatment goals. In this study, the correlation between the number of treatments showed no or minimal correlation with clinical outcomes (skin laxity and rate of improvement). However, a significant moderate correlation was reported with BMI, indicating that patients with overweight or obesity would require more RF sessions. Correlation results also showed no significant low and moderate association between demographics (skin type, age and BMI) and clinical outcomes, except for age and the rate of improvement (*r*(28) = 0.37, *p* = 0.04), reflecting that mature patients may demonstrate greater improvement since those patients usually have greater skin laxity and deeper wrinkles. The most relevant factor related to performance outcome was the skin severity, represented as the initial skin laxity. Data analysis reported a significantly high positive correlation between the initial skin laxity grade of the patients and the change in skin laxity grade (*r*(28) = 0.55, *p* < 0.05), and a significant moderate positive correlation with the rate of improvement (*r*(28) = 0.48, *p* < 0.05). These results confirm the intricacy of RF treatments, which should be assessed individually since the patient's condition influences the clinical outcomes.

The current study acknowledges several limitations that may impact the interpretation of its findings. Although the assessed population included 33 patients, the sample size is small and lacks a control group. The participant demographic was notably homogeneous, primarily female subjects with II–IV skin type. These facts may restrict the statistical power and generalizability of the research results. The short follow‐up period of 1 month further limits the understanding of long‐term treatment effects and tissue response since long‐term follow‐up was not performed personally by the practitioners. Furthermore, the study did not incorporate objective measurement techniques beyond visual assessment, introducing potential subjective bias. These limitations do not diminish the value of the study but rather highlight the crucial need for more comprehensive and further research on RF devices.

## 5 Conclusions

According to retrospective data collected from three separate clinical sites, animal model ex vivo and in vivo experiments reveal the RF V‐FC handpiece as an effective treatment for facial contouring and circumferential reduction, with a confirmed short‐ and long‐term high safety profile.

## Author Contributions

M.E., E.W., S.R.G., and I.B. conducted the research and contributed to data acquisition. R.V. served as a blind evaluator. O.C.L., S.R.G., and I.B. contributed to the data analysis. All authors were involved in the drafting and revision of the manuscript and gave final approval for the version to be published. Each author has agreed to be responsible for all aspects of the job to ensure that issues relating to the accuracy or integrity of any part of the job are properly investigated and resolved.

## Ethics Statement

All study procedures were conducted in compliance with the 1975 Declaration of Helsinki. The authors confirm that the ethical review committee approval is not necessary, as the study device is an already ce‐marked device.

## Conflicts of Interest

R.V., O.C.L., and I.B. are employed by Sinclair EBD. The authors declare that the study was conducted free of any commercial or financial relationships that could be construed as a potential conflicts of interest.

## Data Availability

The data that support the findings of this study are available on request from the corresponding author. The data are not publicly available due to privacy or ethical restrictions.
